# Customers’ Knowledge, Attitude, and Practices towards Food Hygiene and Safety Standards of Handlers in Food Facilities in Hanoi, Vietnam

**DOI:** 10.3390/ijerph15102101

**Published:** 2018-09-25

**Authors:** Anh Tuan Le Nguyen, Bach Xuan Tran, Huong Thi Le, Xuan Thanh Thi Le, Khanh Nam Do, Hoa Thi Do, Giang Thu Vu, Long Hoang Nguyen, Carl A. Latkin, Cyrus S. H. Ho, Roger C. M. Ho

**Affiliations:** 1 Institute for Preventive Medicine and Public Health, Hanoi Medical University, Hanoi 100000, Vietnam; dennguyenle@gmail.com (A.T.L.N.); lethihuong@hmu.edu.vn (H.T.L.); lethithanhxuan@hmu.edu.vn (X.T.T.L.); donamkhanh@hmu.edu.vn (K.N.D.); dothihoa1954@yahoo.com (H.T.D.); 2 Bloomberg School of Public Health, Johns Hopkins University, Baltimore, MD 21205, USA; carl.latkin@jhu.edu; 3 Institute for Global Health Innovations, Duy Tan University, Da Nang 550000, Vietnam; giang88.ighi@gmail.com; 4 Center of Excellence in Behavioral Medicine, Nguyen Tat Thanh University, Ho Chi Minh City 700000, Vietnam; longnh.ph@gmail.com; 5 Department of Psychological Medicine, National University Hospital, Singapore 119074, Singapore; cyrushosh@gmail.com; 6 Department of Psychological Medicine, Yong Loo Lin School of Medicine, National University of Singapore, Singapore 119228, Singapore; hocmroger@yahoo.com.sg

**Keywords:** food safety, food handlers, customers, knowledge, attitude, practices

## Abstract

Efforts to prevent foodborne illnesses in food facilities require sufficient knowledge on hygiene and safety standards from both food processors and customers. However, studies about knowledge, attitude, and practices of customers towards these issues are constrained. This study explored the knowledge, attitude, and practices (KAP) of customers regarding the practices of food facilities as well as potential associated factors. A cross-sectional survey was conducted in Hanoi from September to October 2015. Questions about knowledge, attitude, and practice towards food hygiene and safety were asked, alongside sociodemographic characteristics. Multivariate Tobit regression was used to identify the associated factors with the KAP. Among 1740 customers, the highest mean score of 98.4 (SD = 10.1) was found in knowledge about practices with raw and cooked food, following by knowledge about environmental practices when processing food (mean = 93.1, SD = 17.3), and knowledge about environmental requirements when processing food (mean = 33.3, SD = 33.3). Most of customers considered the processing and selling of hygienic meals without leaving any food overnight as the most important feature for food facilities (73.8%). About 63.2% of participants chose not to report food safety violation by facilities to authorities. The higher score of knowledge was found in groups of people who were not single, had college/university or higher education, and had specific criteria when choosing their places to eat. These findings imply the need for enhancing customers’ protection systems, the capability of inspecting and supervising the food processing progress by local authorities, and the awareness of customers about the environmental requirements of food facilities.

## 1. Introduction

Foodborne diseases, which result from consuming food having contaminants of viruses, bacteria, parasites, chemicals, and allergens, have been a seemingly never-ending threat to public health and a significant hindrance to the development of socio-economy worldwide [1]. According to the World Health Organisation (WHO), almost 1 in every 10 people in the world get sick after eating contaminated food, and 420,000 people die every year [2]. In low and middle-income countries, foodborne illnesses have a tendency to increase due to the surge witnessed in the consumption of risky foods, namely farm animals, fish products, and fresh produce [3]. The continent of Africa and Southeast Asia have been deemed to have the highest rates of incidence and mortality associated with foodborne diseases [4].

In Vietnam, the Ministry of Health reported that there were 677 incidents of food poisoning that affected more than 21,000 people over the period of 2011 to 2014 [5]. These numbers might just be an underestimation, since there are potentially many cases unrecognized, un-investigated or unreported in the communities [6]. The long-term effects of absorbing chemicals in contaminated food, which is generally believed in the community as possible links to getting cancers, have not been properly covered in Vietnam [7].

In order to ensure food safety, the Vietnamese Government has attempted to strengthen food safety management systems, increase assurance in food production, trading and processing via the implementation of the Umbrella Law on Food Safety (FSL), along with the establishment of an inter-ministerial steering committee for creating the state management of food safety and many policies for enhancing production and market development for safe foods [8]. However, the effectiveness of such efforts may be undermined by existing economic and developmental issues such as legislation, infrastructure, enforcement mechanisms, and people’s awareness, which occur in Vietnam and other low- and middle-income countries as well [9]. Moreover, the country has long possessed a culture where street food plays an important role, while the majority of foodborne disease outbreaks were reported at the food facilities instead of home settings [6]. Although the main responsibility of ensuring food safety may be that of food producers, manufacturers, and traders, it has been argued that consumers should also be proactive and take preventive cautions, adhering to food safety practices in order to protect themselves from the risks of foodborne illnesses [10]. Literature looking into the customer’s knowledge, perception, and behaviour of food hygiene and safety of food facilities in Vietnam has been limited. One study in Ho Chi Minh City reported that 17.5% consumers had poor food safety knowledge levels [11]. Due to the lack of evidence-based information about consumers’ knowledge, attitudes, and practices (KAP) of food safety in Vietnam, we conducted this study in order to explore the KAP of customers regarding the practices of food facilities as well as potential associated factors.

## 2. Materials and Methods

### 2.1. Study Setting, Sample Size, and Sampling Method

We conducted a cross-sectional survey in Hanoi, the capital of Vietnam, from September to October 2015. Hanoi is one of the greatest metropolitans in Vietnam, with approximately 7.7 million residents within 30 districts and 584 communes [12]. We first listed all communes within 29/30 districts and then randomly selected 176 communes. In each commune, we prepared the list of food facilities managed by the local authorities and randomly selected ten of them. The eligible criteria for food facilities included: (1) offering food services; and (2) registered with the local authorities.

In each food facility, we purposively selected one customer visiting the facility after our data collectors. The participants were invited to enrol in the study if they met the following criteria: (1) visiting food facilities in the period of the study; (2) aged 15 and above; and (3) agreed to participate in the study. We intended to recruit 1760 participants, and after cleaning the data, 1740 customers (98.9%) were appropriate for analysis.

### 2.2. Measures and Instruments

Data were collected through face-to-face interviews conducted with trained post-graduate students from Hanoi Medical University, using a structured questionnaire.

The collected information consisted of socio-economic characteristics (i.e., gender, education attainment, marital status, employment, and household income); habits in visiting food facilities (e.g., frequency of visiting food facilities, feeling secure when eating out, and criteria when choosing food facilities); what customers considered the most important feature for a food facility; and whether they reported to the local agency when spotting violations of food hygiene regulations at food facilities. 

In terms of knowledge about hygienic practices of handlers in food facilities, we asked customers to report their perceptions about (1) use chopsticks/tongs/knives for raw and cooked food; (2) necessity of having trash and clean water; (3) hygienic requirements for water, trash, and food handlers; and (4) effects of cleaning food processing places or keeping food in glass cabinets. 

### 2.3. Statistical Analysis

Data were analysed using STATA 12.0 (StataCorp, LP, College Station, TX, USA). Exploratory factor analysis (EFA) was employed to explore the construct validity of the questionnaire. In order to extract factors, we used principal component analysis with a threshold of an eigenvalue of 1.0, which is identified by using the screen test. Orthogonal Varimax rotation with Kaisers’ normalization was used to re-construct items in the questionnaire in order to alleviate the interpretability of the results. A threshold of 0.35 was used for factor loadings. In addition, based on the nature of items and the overarching dimension, we performed a cross-loading for one item and allocated to the appropriate domain. After conducting EFA, three customers’ knowledge domains of environmental requirements when processing food, practices with raw and cooked food, and environmental practices when processing food were identified, which had Cronbach’s alpha of 0.758, 0784 and 0.453, respectively.

With each correct answer scoring 1 point, we calculated the sum of each domain and transformed to a 100-point scale, with 0 as “No knowledge” and 100 as “Full knowledge”. Tobit regression (or censored regression) was used to determine associated factors with the score of each domain and the total score of knowledge. A *p*-value < 0.05 was considered statistical significance.

### 2.4. Ethics Approval

The study protocol was approved by the Institutional Review Board (IRB) of the Hanoi Department of Health and the Institute for Preventive Medicine and Public Health (Project identification code 06/CCATVSTPHN).

## 3. Results

Table 1 shows that the mean age was 34.6 (SD = 12.9). Most of the respondents were female (61.9%), living with a spouse/partner (64.3%), having more than a high school education (55.9%), were office workers (30.6%), and frequently using catering services (68.6%). The street vendor was the most common type of food facility selected (48%).

Table 2 reveals that most people (98.7%) were aware of the necessity of using chopsticks or tongs in picking up cooked food, while only less than 20% fully knew the hygienic criteria for a trash can. The highest mean knowledge score of the respondents was found in practices with raw and cooked food (98.4), while the lowest score was recorded in the domain of environmental requirements when processing food (33.0).

Table 3 shows that most of the respondents chose food facilities if those facilities were clean and certified by the food safety authorities (71.0%), and 58.4% chose the facilities with clean places for preparing food. The most important feature for food facilities was the freshness and cleanliness of the food served (73.8%). However, 63.2% chose not reporting to the local agency if finding food facilities violating food hygiene and safety regulations.

Table 4 reveals that the total score of knowledge was found positively associated with people who were not single and those who attended college/university or higher education. Having specific criteria when choosing their places to eat was also a factor that correlated with the increase in total knowledge score. Meanwhile, blue-collar workers and people belonging to the poorest household income quintiles tended to have lower knowledge score than white-collar workers and those belonging to rich income groups. Moreover, those who considered reasonable price to be the most important feature for food facilities selection also had lower scores of knowledge than those who selected food facilities that sold meals with good hygiene and did not store any food overnight.

## 4. Discussion

Our study provides an insight into the knowledge, attitude, and practices toward food hygiene and safety standards among customers in food facilities. The study found that while the majority of customers had good knowledge about practices with raw and cooked food, as well as environmental practices when processing food, only one-third of the respondents demonstrated sufficient understanding about the environmental requirements for such practices. The high percentage of customers with knowledge on raw food handling found in this study was in line with a study by Redmond et al. (2004) which indicated that more than 90% of consumers understood the necessity of separating kitchen cutlery for raw and ready-to-eat foods [13]. Another study on the Irish population also found that only 3% of the consumers reused the knife previously used for raw meat cutting [14]. On the other hand, even though the majority of respondents understood the necessity of having trash bins located inside the facilities and the source of water should be tested on an annual basis, only 18.7% of them knew the hygienic criteria for a trash can, while 36.5% were able to list the hygienic requirements for water used for cooking. We suppose the insufficient knowledge regarding this issue among our customers is due to their belief that it was the responsibility of providers to deliver food services with clean and basic amenities [15]. Moreover, they might depend on visual indications and/or the overall cleanliness of the food facilities to decide the degree of safety and quality of the meal [16].

With regards to the criteria for choosing the food facilities, the cleanliness of facilities and food processing places was the most common factor cited by our participants. Meanwhile, customers reported perceiving the hygiene and safety of food as the most important factor of a food facility. This finding was comparable to a study conducted in Canada that reported the safety of meal was a predominant trait affecting the decision of dining outside, while the most important indicators of the restaurant’s safety were reported to be the cleanliness of the kitchen, utensils, dining area, and restrooms [17]. Another study on Americans having meals at Asian and Mexican restaurants also found the cleanliness of the kitchen to be the most important feature signalling the food safety [18]. Nonetheless, other criteria like appearance, taste, ingredients, price, previous experience, location, and the reputation of the food facilities [19] were also considered by customers when deciding which food facilities they patronized. This statement was reaffirmed by our results as almost 20% of the people considered reasonable price was the most important feature of a food facility, while 7% regarded the attitudes and qualities of the sellers to be the most crucial factor.

In terms of customers’ complaints to local agencies, only 36.8% of the consumers reported back to the authorities when the food facilities violated the hygiene and safety regulations. Some stated that they would forgive the facilities if the problems were handled professionally [18]. It was also generally believed that reporting to the authorities would not be worthy of the time, as perceived lack of manpower would hindered the effectiveness of governmental efforts in addressing such safety violations anyway [18]. The annual report from Vietnam Competition & Consumer Authority in 2017 showed that the hotline for supporting and protecting the consumers could only handle 54% of the total customers’ calls [20]. In addition, it has been argued that the consumers were most likely to doubt the guarantees to offer the food safety from authorities and market parties via their policies and brandings [21]. A study in Hanoi on the consumption of vegetables showed that 93% of the urban population was concerned about the safety of the food they consumed and almost 30% of them distrusted the food safety certificate issued by the government [22].

Our study also found that customers’ knowledge associated with the hygienic practices of food handlers within the food service facilities varied among different sub-groups. For instance, white-collar workers were much more knowledgeable about the food handlers’ practices in the eating outlets than those who were retired or doing manual labour jobs. These results were in line with a study by Behren et al. (2015) which found that those who worked for a company in the communication sector were more aware of the food quality than those who worked for the low-income public supported program [16]. Moreover, those who deemed price as the most important factor in choosing food facilities were likely to have lower knowledge than people considering the hygiene and safety of food to be the most deciding one. It could be due to the fulfilment of basic food requirements that customers started to choose restaurants with better food hygiene and the financial disadvantages while limited access to various kinds of food also influenced the customers’ choices of food facilities [23].

Several implications can be drawn from this study. Firstly, the system of protecting the customers should be improved, especially the hotline for supporting and protecting the customers. As the most important factor of choosing the food facilities was the freshness and cleanliness of the food served and customers tended to believe their own judgement on the food safety of the place of dining rather than others’ point of view and official authorities [17], the local agencies need to strengthen their inspection abilities and invest more on manpower to better supervise the food processing progress in the food facilities. The act of tightening the food safety certification process is also essential and should be focused on by the authorities in order to gain trust from the community and heavier penalties should be imposed for violating the food safety regulations and displaying fake certifications. For a longer-term view, stakeholders of those food-selling businesses need to be empowered and motivated, through perhaps educational campaigns, to realize that their self-regulation under the control of authorities is more effective and efficient in the long term [7]. Apart from that, efforts should be made to raise the customers’ awareness of the hygienic requirements of equipment used when processing, containing, and disposing of food in food facilities.

This study had several limitations. Its cross-sectional and self-reported setting meant that it potentially suffered from participants’ recall errors, while the ability to evaluate the causal relationship between the socio-demographic factors and customers’ knowledge would be limited. Furthermore, the low Cronbach’s alpha in some indicators of the study implied that further efforts should be made to improve internal consistency. There needs to be future studies with a larger sample size and enhanced measurement instruments.

## 5. Conclusions

In conclusion, our study found that customers possessed sufficient knowledge of food handling practices, but rather low understanding of the facility requirements of processing, containing, and disposing of food. The most important criteria for them to choose a food facility were the freshness and cleanliness of the meal served; however, the majority of respondents were not willing to report violations of food hygiene and safety by facilities to the authorities. These findings imply the need for enhancing the system of protecting customers along with the capability for food processing inspection and supervision by local authorities, as well as raising the people’ awareness of environmental requirements for food facilities.

## Figures and Tables

**Table 1:** Socioeconomic characteristics of respondents.

Characteristics	Male		Female		Total		*p*-Value
	*n*	%	*n*	%	*n*	%	
**Total**	663	38.1	1077	61.9	1740	100.0	
**Marital status**							
Single	279	42.2	312	29.0	591	34.0	*p* < 0.01
Live with spouse/partner	372	56.2	744	69.2	1116	64.3	
Divorced/widow	11	1.7	19	1.8	30	1.7	
**Education**							
Illiterate	4	0.6	4	0.4	8	0.5	0.07
Primary school	13	2.0	22	2.1	35	2.0	
Secondary school	69	10.6	112	10.5	181	10.5	
High school	212	32.4	323	30.2	535	31.1	
College, university	330	50.5	589	55.1	919	53.3	
After graduation	26	4.0	19	1.8	45	2.6	
**Employment**							
Students	117	17.7	192	17.8	309	17.8	*p* < 0.01
Worker	164	24.8	140	13.0	304	17.5	
Office workers	186	28.1	345	32.1	531	30.6	
Retire	53	8.0	74	6.9	127	7.3	
Housewife	15	2.3	218	20.3	233	13.4	
Unemployment	22	3.3	14	1.3	36	2.1	
Other jobs	105	15.9	93	8.6	198	11.4	
**Frequently using catering services**							
Yes	488	74.7	690	64.9	1178	68.6	*p* < 0.01
No	165	25.3	374	35.2	539	31.4	
**Feeling secure when eating out**							
Yes	232	35.1	317	29.5	549	31.7	0.04
No	126	19.1	205	19.1	331	19.1	
Depending on each food facility	303	45.8	551	51.4	854	49.3	
**Type of current food facility**							
Fast food	138	21.6	254	24.4	392	23.3	0.44
Restaurant	155	24.2	224	21.5	379	22.5	
Street vendor	307	48.0	501	48.1	808	48.0	
Others	40	6.3	63	6.1	103	6.1	
	**Mean**	**SD**	**Mean**	**SD**	**Mean**	**SD**	***p*-Value**
**Age**	34.2	12.7	34.9	12.9	34.6	12.9	0.26

**Table 2:** Knowledge about food hygiene and safety among customers in food facilities.

Items	% Having Correct Answers	Knowledge about Environmental Requirements When Processing Food	Knowledge about Practices with Raw and Cooked Food	Knowledge about Environmental Practices When Processing Food
Use chopsticks or tongs to pick cooked food	98.7		0.670	
Use separate tongs for raw and cooked foods	98.5		0.825	
Use separate knives for raw and cooked foods	97.8		0.859	
Be able to list the necessity of having a trash	96.4			0.813
Processing food at least 60 cm from the ground	93.7			0.830
Water should be tested once a year	89.2			0.398
Be able to list the positive effects of containing food in glass cabinets	44.3	0.701		
Be able to list hygienic requirements for water used for cooking	36.5	0.612		
Be able to list hygienic requirements for people cooking food	35.9	0.751		
Be able to list positive effects of cleaning or keeping hygiene in places where food is processed	29.4	0.801		
Be able to list hygienic requirements for a trash	18.7	0.690		
Cronbach’s alpha		0.758	0.784	0.453
Mean		33.0	98.4	93.1
SD		33.0	10.1	17.3

**Table 3:** Criteria when choosing food facilities among customers.

Characteristics	Male		Female		Total		*p*-Value
	*n*	%	*n*	%	*n*	%	
**Criteria when choosing food facilities**							
Clean facilities, with a certificate of food hygiene and safety	459	69.9	771	71.7	1230	71.0	0.41
Clean-looking facilities, no need to have a certificate of food hygiene and safety	210	32.1	358	33.3	568	32.9	0.61
Having clean food processing places	352	53.7	658	61.2	1010	58.4	<0.01
Separating raw food and cooked food	260	39.8	483	45.0	743	43.0	0.04
No, leave food on the ground	213	32.6	366	34.1	579	33.5	0.53
Use drugs on mouse and cockroach killing in the food processing places	48	7.4	90	8.4	138	8.0	0.45
**The most important feature for a food facility**							
Food processing and selling during a day with good hygiene and safety	448	70.4	783	75.8	1231	73.8	0.03
Have reasonable prices	122	19.2	182	17.6	304	18.2	
Sellers have good attitudes and are professional	57	9.0	59	5.7	116	7.0	
Others	9	1.4	9	0.9	18	1.1	
**Reporting to the local agency if finding food facilities violating food hygiene and safety regulations**							
Yes	261	39.9	375	34.9	636	36.8	0.04
No	394	60.2	699	65.1	1093	63.2	

**Table 4:** Factors associated with knowledge of customers regarding hygienic practices of food handlers in food facilities.

Characteristics	Knowledge about Practices with Raw and Cooked Food		Knowledge about Environmental Requirements When Processing Food		Knowledge about Environmental Practices When Processing Food		Total Score of Knowledge	
	Coef.	95%CI	Coef.	95%CI	Coef.	95%CI	Coef.	95%CI
Age							−0.04	−0.12; 0.04
Gender (Female vs. Male)			4.10	−1.01; 9.21				
Marital status (vs. Single)								
Having spouse/partner			5.82 **	0.50; 11.14			2.06 **	0.12; 4.00
Education attainment (vs. Illiterate)								
Secondary school			−7.03	−15.56; 1.51				
High School							2.02 *	−0.32; 4.35
College, University			5.63 **	0.07; 11.20			4.10 ***	1.74; 6.46
Post-graduate					−24.60 *	−52.73; 3.53		
Occupation (VS. White−collar workers)								
Blue-collar worker	−48.51 **	−90.84; −6.18	−8.18 **	−15.09; −1.27			−2.36 **	−4.66; −0.07
Retirement	−67.82 **	−125.88; −9.76	−9.91 **	−19.25; −0.58			−2.40	−5.85; 1.04
House-makers							1.88	−0.71; 4.47
Household income quintiles (vs. Poorest)								
Poor	55.88 *	−1.83; 113.58						
Middle	34.72	−8.57; 78.01						
Rich	−28.74	−78.90; 21.42	−10.95 ***	−18.87; −3.03			−3.08 **	−5.56; −0.61
Richest			−5.09	−11.78; 1.59			−1.97 *	−4.07; 0.14
Frequently using catering services (Yes vs. No)							−1.18	−2.86; 0.51
Feeling secure when eating out (vs. Yes)								
No	−82.07 ***	−135.81; −28.33	7.97 ***	2.02; 13.91	−8.89	−22.22; 4.45	1.55	−0.39; 3.49
Depending on each food facility	−41.66 *	−87.73; 4.41						
Type of current food facility (vs. Fast food)								
Restaurant	23.77	−12.25; 59.80	−6.65 ***	−11.48; −1.81			−1.37*	−2.90; 0.16
The most important feature of a food facility (vs. Food processing and selling during a day with good hygiene and safety)								
Have reasonable prices	−77.35 ***	−119.47; −35.23	−10.16 ***	−16.86; −3.45	−33.43 ***	−46.63; −20.23	−5.28 ***	−7.32; −3.24
Criteria when choosing food facilities (Yes vs. No)								
Clean facilities, with a certificate of food hygiene and safety	100.91 ***	50.11; 151.71	40.85 ***	34.48; 47.22	38.12 ***	24.37; 51.87	14.27 ***	12.37; 16.18
Clean-looking facilities, no need to have a certificate of food hygiene and safety	62.96 ***	15.50; 110.42	20.13 ***	14.38; 25.88	27.15 ***	13.27; 41.03	7.47 ***	5.65; 9.28
Having clean food processing places	43.05 **	3.84; 82.26	6.22 **	0.90; 11.54	23.67 ***	11.87; 35.48	3.57 ***	1.91; 5.23
Separating raw food and cooked food	56.81 **	10.97; 102.65	19.33 ***	13.69; 24.97			5.98 ***	4.20; 7.75
No, leave food on the ground			23.25 ***	17.43; 29.07	15.98 **	1.98; 29.98	8.28 ***	6.42; 10.13
Use drugs on mouse and cockroach killing in the food processing places					−49.19 ***	−69.57; −28.81		

* *p* < 0.1; ** *p* < 0.5; *** *p* < 0.01.
